# Factors associated with fear of childbirth in pregnant women in a University Hospital in Southern Brazil

**DOI:** 10.1590/1980-220X-REEUSP-2025-0336en

**Published:** 2026-06-12

**Authors:** Priscilla Scucato Minioli, Marina Accioly de Castro, Raíssa Almeida Ramos, Ana Paula Maia Dal Moro, Beatriz Corrêa Crispim, Luiz Felipe Minioli Krul, Sarah Cristina Zanghellini Rückl

**Affiliations:** 1 Universidade Federal do Paraná, Departamento de Tocoginecologia, Curitiba, PR, Brazil.; 2 Universidade Federal do Paraná, Complexo do Hospital de Clínicas, Curitiba, PR, Brazil.; 3 Faculdade Evangélica Mackenzie do Paraná Curitiba, PR, Brazil.; 4 Universidade Federal do Paraná, Departamento de Psiquiatria, Curitiba, PR, Brazil.

**Keywords:** Parturition, Anxiety, Pregnant People, Prenatal Care, Cross-Sectional Studies, Parto, Ansiedade, Gestantes, Cuidado Pré-Natal, Estudos Transversais

## Abstract

**Objective:**

To identify the main fears related to childbirth in pregnant women in a University Hospital in Southern Brazil, from 2021 to 2023, according to the motivations for fear.

**Method:**

A cross-sectional study was conducted with pregnant individuals between 28 and 37 weeks of gestation who answered two self-administered questionnaires: the Wijma Delivery Expectancy Questionnaire (Version A) and a researcher-designed instrument on specific childbirth fears. Based on scores, participants were classified into four levels of fear (low to very high). Descriptive statistics were applied, and differences in the distribution of specific childbirth-related fears across levels of fear were analyzed using the chi-square or Fisher’s exact tests (p ≤ 0.05).

**Results:**

The sample included 334 participants. Moderate fear was most prevalent (44.9%), with tokophobia occurring in 4.8%. High fear levels were significantly associated with fear of panicking, losing control, not knowing labor duration, and intense pain (p < 0.001).

**Conclusion:**

These findings demonstrate that the unpredictability of childbirth and the perception of lack of control over the situation are key factors in intensifying childbirth fear, emphasizing that incorporating psychological support and a structured health education program into prenatal care may help reduce fear and improve maternal and neonatal outcomes.

## RESUMO

**Objetivo:**

Identificar os principais medos relacionados ao parto em gestantes em um Hospital Universitário do Sul do Brasil, de 2021 a 2023, de acordo com as motivações para o medo.

**Método:**

Foi realizado um estudo transversal com gestantes entre 28 e 37 semanas de gestação, que responderam a dois questionários autoaplicáveis: o Wijma Delivery Expectancy Questionnaire (Versão A) e um instrumento desenvolvido pelos pesquisadores sobre medos específicos relacionados ao parto. Com base nas pontuações, os participantes foram classificados em quatro níveis de medo (baixo a muito alto). Foram aplicadas estatísticas descritivas e as diferenças na distribuição de medos específicos relacionados ao parto, de acordo com os níveis de medo, foram analisadas utilizando os testes qui-quadrado ou exato de Fisher (p ≤ 0,05).

**Resultados:**

A amostra incluiu 334 participantes. O medo moderado foi o mais prevalente (44,9%), seguido pela tocofobia em 4,8% dos casos. Níveis elevados de medo estiveram significativamente associados ao medo de pânico, perda de controle, desconhecimento da duração do trabalho de parto e dor intensa (p < 0,001).

**Conclusão:**

Esses achados demonstram que a imprevisibilidade do parto e a percepção de falta de controle sobre a situação são fatores-chave que intensificam o medo do parto, enfatizando que a incorporação de apoio psicológico e um programa estruturado de educação em saúde no pré-natal pode ajudar a reduzir o medo e melhorar os resultados maternos e neonatais.

## INTRODUCTION

Fear of childbirth is described and defined as a persistent feeling of fear about giving birth that limits daily and social activities, as originally proposed by Marcé in 1858, and cited in apud Hofberg and Brockington^(1)^, significantly influencing the pregnant woman’s journey and her birthing process. If undiagnosed, it can result in a sense of loneliness and lack of support, affecting not only the woman’s mental health but also the baby’s health^(2)^. Its intensity can range from insignificant to extreme fear^(3)^, often leading the pregnant woman to believe that her own life or the baby’s life is at risk^(4)^.

The prevalence of childbirth fear varies worldwide, affecting between 3.7% and 43% of women^(5)^. The reasons for these variations are unknown but may be influenced by cultural differences in women’s perceptions and expectations about childbirth^(6)^, as well as by the quality of medical care, psychological and obstetric factors, and the level of social support^(7)^.

The reasons for women’s fear of childbirth are varied and multifactorial, and may be associated with a combination of predisposing factors, such as anxiety, depression, and other psychiatric disorders, conflicts in interpersonal relationships, a history of sexual abuse, and a traumatic previous childbirth experience^(8,9)^. The context or environment in which women give birth and their interactions with healthcare professionals also influence childbirth fear^(10)^. Many pregnant women worry about the obstetric team’s hostility, the possibility of being left alone during labor, and the lack of involvement in decision-making^(11)^.

Factors related to the childbirth event, such as intolerance of uncertainty, childbirth self-efficacy^(12)^, pain during the first stage of labor, feelings of helplessness, unmet expectations, and the need for medical interventions, are significant predictors of perceiving childbirth as a traumatic event^(10)^. Parity also influences the causes of childbirth fear, as nulliparous women tend to fear the unknown, pain, and loss of control, whereas multiparous women are more likely to experience fear arising from previous traumatic experience^(13)^.

In clinical practice, identifying women’s suffering from fear of childbirth is challenging due to discrepancies in both the definition and measurement of this condition^(3,14)^. Although there are no standard criteria for diagnosing fear of childbirth, the most widely used tool is the Wijma Delivery Expectancy Questionnaire (Version A) (W-DEQ(A)), developed by Wijma, Wijma, and Zar in 1998^(3)^. The questionnaire score ranges from zero to 165, and the higher the score, the more severe the fear of childbirth, with scores ≥ 100 considered tokophobia, a term characterized as “an irrational dread of childbirth” due to severe distress, with thoughts and behaviors aimed at avoiding childbirth^(1,15)^. In Brazil, a study with 125 pregnant women between 28 and 36 weeks found that 12% of them scored ≥ 85 on the W-DEQ(A), indicating very high childbirth fear, while 5.6% scored ≥ 100, indicating tokophobia, with an association between childbirth fear and anxiety symptoms, depressive symptoms, and poor social support^(16)^. However, there remains a need to further explore how specific fear-related motivations are distributed across different levels of childbirth fear in larger samples and real- world clinical settings within the Brazilian population context.

The volume of publications on fear of childbirth has increased nearly 30-fold over the past two decades^(17)^, but this topic has only recently begun to be investigated in Brazil. Understanding childbirth-related fears and their associated factors is crucial to guide psychological and educational interventions, enhance the quality of obstetric care, and promote a more positive childbirth experience. In order to contribute to a broader understanding of this phenomenon within the Brazilian population context, this study aims to identify the main fears related to childbirth among pregnant women in a University Hospital in Southern Brazil, between 2021 and 2023, according to the motivations for fear.

## METHOD

### Study Design

This is a cross-sectional observational study reported in accordance with the Strengthening the Reporting of Observational Studies in Epidemiology (STROBE) Statement guidelines.

### Population

The study population consisted of pregnant women receiving care between 2021 and 2023, a period chosen based on the feasibility of data collection and to ensure an adequate and representative sample.

### Local

The study was conducted at the Maternity Ward of the Universidade Federal do Paraná Hospital Complex, a tertiary healthcare center and a referral facility for high-risk pregnancies within the Brazilian Public Health System (*SUS*), which also provides care for low-risk pregnancies. The hospital is a university institution located in Curitiba, Paraná. This setting was chosen due to its relevance as a public teaching and research hospital, presenting a diverse patient population and representing the reality of the public healthcare system.

### Selection Criteria

Participants were recruited through convenience sampling while attending prenatal consultations, undergoing prenatal exams, or participating in prenatal education workshops.

Pregnant women over the age of 18, Brazilian, literate, between 28 and 37 weeks of gestation, and able to understand and answer the questionnaires were included in the study. The choice of this gestational range was based on the fact that, during the third trimester, emotions and fears become more concrete, making tokophobia more evident and measurable. Participants who did not meet the inclusion criteria, as well as those who submitted an incomplete W-DEQ(A), questionnaire were excluded from the study.

### Sample Size Calculation

Considering that the study site is a regional referral center for high-risk pregnancies, it was necessary to balance the sample between two risk strata—high-risk and low-risk pregnancies—to ensure adequate representation. The sample size was estimated assuming an infinite population, using a stratified random sampling design^(18)^. For the calculation, an expected prevalence of 10%, derived from Nilsson et al.^(2)^, was used as the reference parameter. The effect size was defined as 4%, corresponding to half the difference between the values reported in that study (14.8–6.3). A 95% confidence level and a 5% margin of error were applied. Based on these parameters, the minimum required sample size was 217 participants. To account for an anticipated 30% loss, the final sample included a minimum of 283 pregnant women, equally distributed between the two strata.

### Data Collection

After receiving standardized written and verbal instructions provided by the research team to ensure understanding and consistency in responses, the participants answered two self-administered questionnaires: the W-DEQ(A)^(3)^ and another onedeveloped by the authors to assess possible fears related to childbirth. As no validation of the W-DEQ (A) scale for Brazilian pregnant women was identified, the scale was translated into Brazilian Portuguese and back-translated into English. The back-translated version showed a Cronbach’s alpha of 0.71, indicating a good internal consistency with the original version. For the W-DEQ(A) questionnaire, results between 0 and 37 were classified as “low fear”, scores between 38 and 65 as “moderate fear”, scores ≥ 65 as “high fear”, scores ≥ 85 as “very high fear”, and scores ≥ 100 were considered tokophobia^(2,19)^. The questionnaire developed by the authors was constructed based on a comprehensive literature review on the topic. Although the instrument was not formally validated, a pilot test was conducted with a small group of pregnant women to assess clarity and understanding, and minor adjustments were made based on their feedback.

### Data Analysis and Treatment

For statistical analysis, an initial descriptive analysis of absolute and relative frequencies of qualitative variables was performed. To evaluate differences in the distribution of motivations for fear across levels of childbirth fear, proportions were compared using the chi-square test. When the assumptions of the chi-square test were not met, Fisher’s exact test was applied. A 5% significance level was adopted, and all analyses were conducted using R software version 4.2.1^(20)^. Differences were considered statistically significant for p-values ≤ 0.05.

### Ethical Aspects

The study was approved by the Research Ethics Committee of the Hospital Complex of the Universidade Federal do Paraná (approval number: 5.578.110). All procedures were conducted in accordance with institutional guidelines, and the signed informed consent was obtained from all participants prior to the study conduction.

## RESULTS

A total of 314 participants were enrolled in the study. The participants had a mean age of 29 ± 5.9 years. Their sociodemographic and obstetric characteristics are summarized in Table 1.

**Table 1:** Sociodemographic and obstetric characteristics of the study participants at the Maternity Ward of the Universidade Federal do Paraná Hospital Complex – Curitiba, Brazil, from 2021 to 2023.

Variable	N (%)
**Sociodemographic profile**	
Age (N = 334)	
Up to 30 years	196 (58.7)
31 to 40 years	128 (38.3)
Over 40 years	10 (3.0)
Ethnicity (N = 229)	
White	166 (72.5)
African descendant (mixed/Black)	50 (21.8)
Asian	11 (4.8)
Indigenous	2 (0.9)
Marital Status (N = 334)	
Single	91 (27.2)
Married/common-law marriage	240 (71.9)
Divorced	2 (0.6)
Widowed	1 (0.3)
Educational Level (N = 334)	
Incomplete primary education	23 (6.9)
Complete primary education	47 (14.1)
Secondary education	202 (60.5)
Higher education	62 (18.6)
Source of Income (N = 229)	
No income	53 (23.1)
Salaried employee	105 (45.9)
Retired or pensioner	2 (0.9)
Self-employed	47 (20.5)
Other	22 (9.6)
**Obstetric profile**	
First pregnancy (N = 334)	115 (34.4)
Planned pregnancy (N = 333)	123 (36.9)
Desired Pregnancy (N = 227)	169 (74.4)
High risk pregnancy (N = 334)	262 (78.4)
Previous Miscarriage/Fetal Death/Stillbirth (N = 334)	84 (25.1)
History of Sexual and/or Physical Violence (N = 334)	35 (10.5)
Psychiatric Treatment (N = 229)	51 (22.3)
Previous Birth Experience (N = 209)	
Extremely positive	27 (12.9)
Very positive	28 (13.4)
Positive	70 (33.5)
Regular	42 (20.1)
Negative	23 (11.0)
Very negative	8 (3.8)
Extremely negative	11 (5.3)

N: absolute frequency; %: relative frequency in percentage.

The mean W-DEQ(A) score was 59.65 (SD ± 23.41), with scores ranging from 5 to 136. Table 2 presents the stratification of fear levels, indicating that tokophobia (W-DEQ(A) ≥ 100) was observed in 4.8% of participants.

**Table 2:** Frequency of childbirth fear scores among pregnant women at the Maternity Ward of the Universidade Federal do Paraná Hospital Complex – Curitiba, Brazil, between 2021 and 2023.

Variable	N (%)
Fear Score (N = 334)	
Low	52 (15.6)
Moderate	150 (44.9)
High	88 (26.3)
Very High	44 (13.2)
Fear Score (N = 334)	
< 100	318 (95.2)
≥ 100	16 (4.8)

N: absolute frequency; %: relative frequency in percentage.

Comparison of different levels of childbirth fear with specific fear-related motivations showed that higher levels of childbirth fear were associated with greater proportions of certain fear-related motivations. Specifically, fears related to panicking, losing control, intense labor pain, prolonged labor, and uncertainty regarding labor duration were more frequent among women with high and very high levels of fear, accounting for the overall statistical significance observed (p < 0.001) (Table 3).

**Table 3:** Comparison between childbirth fear scores and fear-related motivations among pregnant women at the Maternity Ward of the Universidade Federal do Paraná Hospital Complex – Curitiba, Brazil, between 2021 and 2023.

Variable		N (%)	Low N (%)	Moderate N (%)	High N (%)	Very High N (%)	p-value*
Fear of panicking?	Yes	144 (43.1)	8 (15.4)	46 (30.7)	57 (64.8)	33 (75.0)	<0.001
Fear of losing control of the situation?	Yes	185 (55.4)	13 (25.0)	81 (54.0)	57 (64.8)	34 (77.3)	<0.001
Fear that sexual life will be negatively affected after childbirth?	Yes	49 (14.7)	1 (1.9)	20 (13.3)	19 (21.6)	9 (20.4)	0.009
Fear of labor pain?	Yes	241 (72.6)	25 (49.0)	106 (70.7)	72 (82.8)	38 (86.4)	<0.001
Fear of medical interventions (such as episiotomy, anesthesia)?	Yes	170 (51.0)	19 (36.5)	70 (47.0)	56 (63.6)	25 (56.8)	0.009
Fear of perineal laceration?	Yes	195 (58.7)	20 (38.5)	90 (60.0)	53 (61.6)	32 (72.7)	0.005
Fear of hemorrhage?	Yes	257 (77.2)	36 (69.2)	114 (76.5)	72 (81.8)	35 (79.5)	0.374
Fear of needing an emergency cesarean section?	Yes	144 (43.5)	17 (33.3)	61 (40.7)	45 (52.3)	21 (47.7)	0.128
Fear of being poorly treated by the obstetric team (doctors and nurses)?	Yes	269 (80.8)	35 (67.3)	120 (80.5)	77 (87.5)	37 (84.1)	0.030
Fear of preterm birth?	Yes	214 (64.1)	28 (53.8)	92 (61.3)	64 (72.7)	30 (68.2)	0.110
Fear of having a very long labor?	Yes	261 (78.4)	29 (55.8)	113 (75.3)	79 (90.8)	40 (90.9)	<0.001
Fear that my child will suffer in some way?	Yes	304 (91.3)	42 (80.8)	137 (91.3)	84 (96.5)	41 (93.2)	0.015
Fear of dying during childbirth?	Yes	223 (67.2)	25 (50.0)	100 (66.7)	67 (76.2)	31 (70.4)	0.018
Fear of being seen by my husband/companion in a state of panic?	Yes	31 (13.5)	3 (7.7)	8 (8.2)	13 (21.0)	7 (23.3)	0.030
Fear of losing composure ("losing control")?	Yes	50 (22.0)	3 (7.7)	16 (16.5)	23 (37.7)	8 (26.7)	0.001
Fear of not having the guarantee that I will be able to have a vaginal birth?	Yes	79 (34.8)	6 (15.4)	31 (32.0)	31 (50.0)	11 (37.9)	0.004
Fear of not noticing an abnormal situation?	Yes	150 (66.1)	18 (47.4)	59 (60.8)	50 (80.6)	23 (76.7)	0.002
Fear of not knowing how long labor will last?	Yes	129 (57.3)	12 (30.8)	53 (54.1)	38 (65.5)	26 (86.7)	<0.001
Fear that my husband/companion will not be able to accompany labor?	Yes	127 (55.5)	17 (43.6)	57 (58.2)	38 (61.3)	15 (50.0)	0.292
Fear that my husband/companion will behave badly?	Yes	28 (12.2)	2 (5.1)	8 (8.2)	14 (22.6)	4 (13.3)	0.023
Fear of the amniotic sac rupturing before hospitalization?	Yes	78 (34.1)	10 (25.6)	30 (30.6)	23 (37.1)	15 (50.0)	0.145
Fear of childbirth occurring before arriving at the hospital?	Yes	143 (62.4)	17 (43.4)	65 (66.3)	39 (62.9)	22 (73.3)	0.045
Fear of being hospitalized before the onset of labor?	Yes	65 (28.4)	9 (23.1)	27 (27.5)	18 (29.0)	11 (36.7)	0.660

N: absolute frequency; %: relative frequency in percentage;

*Pearson's Chi-Square Test or Fisher's Exact Test.

## DISCUSSION

This study identified that fear of childbirth was frequent among the participants. Although a considerable proportion of women presented high or very high fear, moderate levels of fear predominated. Participants’ main concerns were related to pain, loss of control, and uncertainty regarding the duration and progression of labor. These findings reinforce that fear of childbirth is a multifactorial phenomenon influenced by both psychological and obstetric factors, and they highlight the importance of addressing emotional and informational needs during prenatal care.

In the present study, fear of labor pain was one of the most frequent and significant childbirth-related fears, suggesting that pain perception and anticipation play a central role in the experience of childbirth fear. These results are consistent with previous studies, which indicate that pain catastrophizing is relevant to the understanding of fear of childbirth^(21)^. Fear of pain and of having a low pain tolerance have been described as common causes of fear of childbirth, serving as predictors of perceived pain intensity during labor^(22,23)^.

In this context, pain influences pregnant women’s behavior through fear, becoming the source of other aversive emotions and concerns related to childbirth. Unlike pregnancy, which allows for gradual adaptation to changes over a long period, childbirth is an event that induces abrupt and intense changes, symbolized by pain intensity and unpredictability, leading to distress, anxiety, and insecurity^(24)^. Fear of labor pain is strongly associated with fear of pain in general, regardless of parity, and is one of the most common reasons for requesting a cesarean section^(8)^. Additionally, fear may affect cognitive functioning, increase psychophysiological reactivity, and influence how women interpret information and perceive labor pain and its consequences^(22)^.

In this study, participants significantly expressed fear of panicking, losing composure, and losing control of the situation. These fears may reflect a lack of confidence in the childbirth process and the inability to regulate their own behavior during labor^(9,25)^.

Fear of prolonged labor and of not knowing how long it will last were also significant findings in the present study. Such fears may be explained by the perception of unpredictability and uncontrollability during childbirth, factors that also contribute to women’s preference for elective cesarean section, to avoid pain and loss of dignity^(26)^.

The present study identified fear of the child suffering and of a vaginal birth not being guaranteed as relevant childbirth-related fears among participants. Similar findings were reported in a study with 325 nulliparous women in Saudi Arabia^(27)^, which found an association between fear of childbirth and of the inability to give birth or of harming or causing suffering to the baby. Additionally, another study found that women’s fear of being unable to give birth may also be related to lack of confidence in the obstetric team, fear of their own competence, and fear of losing control^(9)^.

Fear of dying during childbirth was also a relevant finding, consistent with previous studies in which it was the most common type of fear experienced during pregnancy, childbirth, and the postpartum period^(9,28,29)^. Such fears may reflect concerns related to maternal safety, trust in care, and perceived vulnerability during childbirth.

Fear of being poorly treated by the obstetric team was also identified as prominent among participants. This result aligns with a study with 100 Swedish women with fear of childbirth, where 73% reported that the most common reason for fear was lack of trust in the obstetric team^(13)^. Similarly, a study conducted in the United States found that complaints were often related to healthcare providers who could worsen fears and increase anxiety during the childbirth process^(25)^.

The present study also found fear of medical interventions significantly associated with fear of childbirth. Similar concerns were reported in a recent Brazilian study with 25 high-risk pregnant women, where the vast majority reported fear associated with obstetric care and medical interventions during labor and delivery^(30)^. These findings highlight the importance of trust, communication, and respectful care in mitigating childbirth- related fears.

Participants also significantly reported fear of perineal laceration and that their sexual life would be negatively affected after childbirth. These findings are consistent with previous studies, in which pregnant women reported fear of genital tract injuries^(13,29)^, as well as research conducted in Türkiye where women feared that perineal injuries could impact their sexual life in the future^(31)^.

In a Japanese study with 11 primigravidae, which explored the qualitative dimensions of childbirth fear, two participants reported fear that something abnormal might happen during labor, and one participant expressed fear that her companion would behave inappropriately^(32)^. These findings align with those of the present study, where fear of not noticing an abnormal situation during labor and of the companion behaving badly were significant.

Fear of being seen by the husband/companion in a state of panic and of childbirth occurring before arriving at the hospital were significant findings among participants in the present study. No relevant literature addressing these specific fears was identified, suggesting that these may represent novel aspects of childbirth fear.

The items fear of hemorrhage, of needing an emergency cesarean section, of preterm birth, that the husband/companion may not be able to accompany labor, of the amniotic sac rupturing before hospitalization, and of being hospitalized before the onset of labor were not significant in relation to very high childbirth fear. This suggests that patients were less afraid of situations that could be managed by the medical team.

The present study highlights the prevalence, intensity, and characteristics of childbirth-related fears among pregnant women receiving care at a tertiary public maternity hospital in southern Brazil. The predominant concerns involved pain, loss of control and uncertainty, emphasizing the need for systematic screening protocols and individualized interventions to support maternal mental health and well-being. Early identification of these fears can improve maternal psychological well-being, enhance birth preparedness, and promote positive childbirth experiences.

From a professional and public health perspective, integrating fear-reduction strategies into routine prenatal care could improve maternal satisfaction and strengthen overall maternal and neonatal outcomes. Such strategies include individualized education, clear and accessible information, emotional support, and encouragement of active participation in birth planning. In addition, fostering familiarity with the maternity setting and building trust in the obstetric team may help reduce unnecessary interventions.

Nevertheless, some limitations must be acknowledged. The present study was conducted in a regional referral center for high-risk pregnancies, which limited the recruitment of women with low-risk profiles during data collection. As a result, the sample is predominantly comprised of high-risk pregnancies within the public healthcare system, which may restrict the generalizability of the findings to women receiving private care or those with low-risk pregnancies. Moreover, given substantial sociocultural diversity found in Brazil, data collected from a single health center may not be representative of the broader population. Additionally, administering the questionnaire during the third trimester—a period commonly associated with heightened anxiety symptoms—combined with data collection during the enforcement of state legislation allowing elective cesarean sections without medical indication, may have influenced participants’ responses, potentially either amplifying or mitigating reported levels of fear.

Notably, the length and complexity of the questionnaire may also have contributed to a reduced sample size, raising concerns regarding its feasibility in non-research settings. Additionally, as data were collected through self-administered questionnaires, nonresponse to certain items may have occurred, resulting in substantial missing data for some variables and potentially restricting a more comprehensive assessment of factors associated with childbirth fear. Future studies should consider strategies to improve data completeness in self-administered instruments and ensure a more robust evaluation of variables.

Furthermore, this study did not include a qualitative approach to explore the subjective dimensions of women’s fears, which could have provided a deeper understanding of their emotional experiences. Future research should aim to identify key questions that allow for rapid assessment of childbirth fear during routine prenatal consultations, and to develop tools that translate the findings into practical indicators capable of guiding clinical interventions in a more accessible and applicable manner.

The use of the questionnaire W-DEQ(A), originally developed in 1998, in the current obstetric context and without a formal validation for the Brazilian pregnant population also represents a limitation of this study. Although a rigorous translation and back-translation process was performed and the instrument demonstrated acceptable internal consistency in the present sample (Cronbach’s alpha = 0.71), this procedure does not replace a full cultural adaptation and validation process. Therefore, the results should be interpreted with caution. Future studies are warranted to validate the W-DEQ(A) for the Brazilian context and to assess its psychometric performance in contemporary obstetric settings.

Despite these limitations, the study offers meaningful contributions to the field by reinforcing the importance of recognizing fear of childbirth as a significant clinical concern. It emphasizes the need for healthcare professionals to incorporate mental health care alongside multidisciplinary approaches within perinatal services, with the aim of improving outcomes for both mothers and their newborns.

## CONCLUSION

The study identified that women with very high childbirth fear experienced greater fear of panicking, of not knowing how long labor will last, of having a very long labor, of losing control of the situation, and of labor pain. These findings demonstrate that the unpredictability of the childbirth process and the perception of lack of control over the situation are key factors in intensifying childbirth fear. The findings highlight the importance of psychological and informational support strategies for pregnant women, aiming to reduce anxiety and strengthen their confidence in the childbirth process.

## Data Availability

The entire dataset supporting the results of this study was published in the article itself.
