# Supplementary material for: Factors associated with fear of childbirth in pregnant women in a University Hospital in Southern Brazil
Source: Rev Esc Enferm USP. 2026 Jun 12;60:e20250336. doi: 10.1590/1980-220X-REEUSP-2025-0336en (PMC13262975; doi:10.1590/1980-220X-REEUSP-2025-0336en)
Supplement: Translation

# Translation: Factores asociados al miedo al parto en mujeres embarazadas en un hospital universitario del sur de Brasil

**DOI:** 10.1590/1980-220X-REEUSP-2025-0336es

**Authors:** Priscilla Scucato Minioli, Marina Accioly de Castro, Raíssa Almeida Ramos, Ana Paula Maia Dal Moro, Beatriz Corrêa Crispim, Luiz Felipe Minioli Krul, Sarah Cristina Zanghellini Rückl

**Affiliations:** 1 Universidade Federal do Paraná, Departamento de Tocoginecologia, Curitiba, PR, Brasil.; 2 Universidade Federal do Paraná, Complexo do Hospital de Clínicas, Curitiba, PR, Brasil.; 3 Faculdade Evangélica Mackenzie do Paraná Curitiba, PR, Brasil.; 4 Universidade Federal do Paraná, Departamento de Psiquiatria, Curitiba, PR, Brasil.

**Keywords:** Parto, Ansiedad, Personas Embarazadas, Atención Prenatal, Estudios Transversales

## Abstract

**Objetivo:**

Identificar los principales temores relacionados con el parto en mujeres embarazadas en un hospital universitario del sur de Brasil, entre 2021 y 2023, según las motivaciones del miedo.

**Método:**

Se realizó un estudio transversal con mujeres embarazadas de entre 28 y 37 semanas de gestación que respondieron a dos cuestionarios autoadministrados: el Wijma Delivery Expectancy Questionnaire (Versión A) y un instrumento diseñado por los investigadores sobre miedos específicos relacionados con el parto. En función de las puntuaciones obtenidas, los participantes fueron clasificados en cuatro niveles de miedo (de bajo a muy alto). Se aplicaron estadísticas descriptivas y se analizaron las diferencias en la distribución de miedos específicos relacionados con el parto según los niveles de miedo mediante las pruebas de chi-cuadrado o la prueba exacta de Fisher (p ≤ 0,05).

**Resultados:**

La muestra incluyó 334 participantes. El miedo moderado fue el más frecuente (44,9%), mientras que la tocofobia se presentó en el 4,8%. Los altos niveles de miedo se asociaron significativamente con el miedo a entrar en pánico, perder el control, no saber la duración del trabajo de parto y el dolor intenso (p < 0,001).

**Conclusión:**

Estos hallazgos demuestran que la imprevisibilidad del parto y la percepción de falta de control sobre la situación son factores clave para intensificar el miedo al parto, subrayando que la incorporación de apoyo psicológico y un programa estructurado de educación para la salud en la atención prenatal puede ayudar a reducir el miedo y mejorar los resultados maternos y neonatales.

## INTRODUCCIÓN

El miedo al parto se describe y define como un sentimiento persistente de temor hacia el parto que limita las actividades cotidianas y sociales, tal como lo propuso originalmente Marcé en 1858, y citado en apud Hofberg y Brockington^(1)^, lo cual influye significativamente en el recorrido de la mujer embarazada y en su proceso de parto. Si no se diagnostica, puede provocar una sensación de soledad y falta de apoyo, lo que afecta no solo a la salud mental de la mujer, sino también a la salud del bebé^(2)^. Su intensidad puede variar desde un miedo insignificante hasta un miedo extremo^(3)^, lo que a menudo lleva a la mujer embarazada a creer que su propia vida o la del bebé están en peligro^(4)^.

La prevalencia del miedo al parto varía en todo el mundo, afectando entre el 3,7 % y el 43 % de las mujeres^(5)^. Se desconocen las razones de estas variaciones, pero pueden estar influenciadas por diferencias culturales en las percepciones y expectativas de las mujeres sobre el parto^(6)^, así como por la calidad de la atención médica, factores psicológicos y obstétricos, y el nivel de apoyo social^(7)^.

Las razones del miedo de las mujeres al parto son variadas y multifactoriales, y pueden estar asociadas a una combinación de factores predisponentes, como la ansiedad, la depresión y otros trastornos psiquiátricos, conflictos en las relaciones interpersonales, antecedentes de abuso sexual y una experiencia traumática en un parto anterior^(8,9)^. El contexto o entorno en el que las mujeres dan a luz y sus interacciones con los profesionales de la salud también influyen en el miedo al parto^(10)^. Muchas mujeres embarazadas se preocupan por la hostilidad del equipo obstétrico, la posibilidad de quedarse solas durante el parto y la falta de participación en la toma de decisiones^(11)^.

Los factores relacionados con el evento del parto, como la intolerancia a la incertidumbre, la autoeficacia en el parto^(12)^, el dolor durante la primera etapa del parto, los sentimientos de impotencia, las expectativas no cumplidas y la necesidad de intervenciones médicas, son predictores significativos de la percepción del parto como un evento traumático^(10)^. La paridad también influye en las causas del miedo al parto, ya que las mujeres nulíparas tienden a temer lo desconocido, el dolor y la pérdida de control, mientras que las mujeres multíparas son más propensas a experimentar miedo derivado de experiencias traumáticas previas^(13)^.

En la práctica clínica, identificar a las mujeres que sufren de miedo al parto es un desafío debido a las discrepancias tanto en la definición como en la medición de esta afección^(3,14)^. Aunque no existen criterios estándar para diagnosticar el miedo al parto, la herramienta más utilizada es el Cuestionario de Expectativas del Parto de Wijma (Versión A) (W-DEQ(A)), desarrollado por Wijma, Wijma y Zar en 1998^(3)^. La puntuación del cuestionario oscila entre cero y 165, y cuanto mayor es la puntuación, más grave es el miedo al parto; las puntuaciones ≥ 100 se consideran tocofobia, un término caracterizado como “un temor irracional al parto” debido a una angustia grave, con pensamientos y comportamientos destinados a evitar el parto^(1,15)^. En Brasil, un estudio con 125 mujeres embarazadas entre las semanas 28 y 36 encontró que el 12 % de ellas obtuvo una puntuación ≥ 85 en el W-DEQ(A), lo que indica un miedo al parto muy elevado, mientras que el 5,6 % obtuvo una puntuación ≥ 100, lo que indica tocofobia, con una asociación entre el miedo al parto y los síntomas de ansiedad, los síntomas depresivos y el escaso apoyo social^(16)^. Sin embargo, sigue siendo necesario explorar más a fondo cómo se distribuyen las motivaciones específicas relacionadas con el miedo a través de los diferentes niveles de miedo al parto en muestras más amplias y entornos clínicos reales dentro del contexto de la población brasileña.

El volumen de publicaciones sobre el miedo al parto se ha multiplicado casi por 30 en las últimas dos décadas^(17)^, pero este tema solo ha comenzado a investigarse recientemente en Brasil. Comprender los miedos relacionados con el parto y sus factores asociados es crucial para orientar las intervenciones psicológicas y educativas, mejorar la calidad de la atención obstétrica y promover una experiencia de parto más positiva. Con el fin de contribuir a una comprensión más amplia de este fenómeno en el contexto de la población brasileña, este estudio tiene como objetivo identificar los principales miedos relacionados con el parto entre las mujeres embarazadas en un hospital universitario del sur de Brasil, entre 2021 y 2023, según las motivaciones del miedo.

## MÉTODO

### Diseño del Estudio

Se trata de un estudio observacional transversal realizado de acuerdo con las directrices de la Declaración sobre el Fortalecimiento de la Presentación de Estudios Observacionales en Epidemiología (STROBE).

### Población

La población del estudio estuvo compuesta por mujeres embarazadas que recibieron atención entre 2021 y 2023, un período elegido en función de la viabilidad de la recolección de datos y para garantizar una muestra adecuada y representativa.

### Local

El estudio se llevó a cabo en la Maternidad del Complejo Hospitalario de la Universidade Federal do Paraná, un centro de atención terciaria y centro de referencia para embarazos de alto riesgo dentro del Sistema Único de Salud (*SUS*) de Brasil, que también brinda atención a embarazos de bajo riesgo. El hospital es una institución universitaria ubicada en Curitiba, Paraná.

Se eligió este entorno debido a su relevancia como hospital público de enseñanza e investigación, que presenta una población de pacientes diversa y representa la realidad del sistema de salud pública.

### Criterios de Selección

Las participantes fueron reclutadas mediante muestreo por conveniencia mientras asistían a consultas prenatales, se sometían a exámenes prenatales o participaban en talleres de educación prenatal.

Se incluyeron en el estudio las mujeres embarazadas mayores de 18 años, brasileñas, alfabetizadas, entre las 28 y 37 semanas de gestación, y capaces de comprender y responder los cuestionarios. La elección de este rango gestacional se basó en el hecho de que, durante el tercer trimestre, las emociones y los miedos se vuelven más concretos, lo que hace que la tocofobia sea más evidente y medible. Las participantes que no cumplían con los criterios de inclusión, así como aquellas que entregaron un cuestionario W-DEQ(A) incompleto, fueron excluidas del estudio.

### Cálculo del Tamaño de la Muestra

Teniendo en cuenta que el centro del estudio es un centro regional de referencia para embarazos de alto riesgo, fue necesario equilibrar la muestra entre dos estratos de riesgo —embarazos de alto riesgo y de bajo riesgo— para garantizar una representación adecuada. El tamaño de la muestra se estimó suponiendo una población infinita, utilizando un diseño de muestreo aleatorio estratificado^(18)^. Para el cálculo, se utilizó como parámetro de referencia una prevalencia esperada del 10 %, derivada de Nilsson et al.^(2)^. El tamaño del efecto se definió como el 4 %, correspondiente a la mitad de la diferencia entre los valores reportados en ese estudio (14,8–6,3). Se aplicó un nivel de confianza del 95 % y un margen de error del 5 %. Basándose en estos parámetros, el tamaño mínimo requerido de la muestra fue de 217 participantes. Para tener en cuenta una pérdida prevista del 30 %, la muestra final incluyó un mínimo de 283 mujeres embarazadas, distribuidas equitativamente entre los dos estratos.

### Recolección de Datos

Tras recibir instrucciones estandarizadas, tanto escritas como verbales, proporcionadas por el equipo de investigación para garantizar la comprensión y la coherencia en las respuestas, las participantes respondieron a dos cuestionarios autoadministrados^(3)^: el W-DEQ(A) y otro desarrollado por los autores para evaluar posibles miedos relacionados con el parto. Dado que no se identificó ninguna validación de la escala W-DEQ(A) para mujeres embarazadas brasileñas, la escala se tradujo al portugués brasileño y se retradujo al inglés.

La versión re-traducida mostró un alfa de Cronbach de 0,71, lo que indica una buena consistencia interna con la versión original. Para el cuestionario W-DEQ(A), los resultados entre 0 y 37 se clasificaron como “miedo bajo”, las puntuaciones entre 38 y 65 como “miedo moderado”, las puntuaciones ≥ 65 como “miedo alto”, las puntuaciones ≥ 85 como “miedo muy alto” y las puntuaciones ≥ 100 se consideraron tocofobia^(2,19)^. El cuestionario desarrollado por los autores se elaboró a partir de una revisión exhaustiva de la literatura sobre el tema. Aunque el instrumento no se validó formalmente, se realizó una prueba piloto con un pequeño grupo de mujeres embarazadas para evaluar la claridad y la comprensión, y se hicieron ajustes menores basados en sus comentarios.

### Análisis y Tratamiento de Datos

Para el análisis estadístico, se realizó un análisis descriptivo inicial de las frecuencias absolutas y relativas de las variables cualitativas. Para evaluar las diferencias en la distribución de las motivaciones del miedo a través de los niveles de miedo al parto, se compararon las proporciones mediante la prueba de chi-cuadrado. Cuando no se cumplían los supuestos de la prueba de chi-cuadrado, se aplicó la prueba exacta de Fisher. Se adoptó un nivel de significación del 5 %, y todos los análisis se realizaron utilizando el software R versión 4,2,1^(20)^. Las diferencias se consideraron estadísticamente significativas para valores de p ≤ 0,05.

### Aspectos Éticos

El estudio fue aprobado por el Comité de Ética en Investigación del Complejo Hospitalario de la Universidade Federal do Paraná (número de aprobación: 5.578.110). Todos los procedimientos se llevaron a cabo de acuerdo con las directrices institucionales, y se obtuvo el consentimiento informado firmado de todas las participantes antes de la realización del estudio.

## RESULTADOS

Se inscribió en el estudio a un total de 314 participantes. La edad media de las participantes fue de 29 ± 5,9 años. Sus características sociodemográficas y obstétricas se resumen en la Tabla 1.

**Tabla 1:** Características sociodemográficas y obstétricas de las participantes del estudio en la Maternidad del Complejo Hospitalario de la Universidade Federal do Paraná – Curitiba, Brasil, de 2021 a 2023.

Variable	N (%)
**Perfil sociodemográfico**	
Edad (N = 334)	
Hasta 30 años	196 (58,7)
De 31 a 40 años	128 (38,3)
Más de 40 años	10 (3,0)
Origen étnico (N = 229)	
Blanco	166 (72,5)
Afrodescendiente (mestizo/negro)	50 (21,8)
Asiático	11 (4,8)
Indígena	2 (0,9)
Estado civil (N = 334)	
Soltero	91 (27,2)
Casado/en unión libre	240 (71,9)
Divorciado	2 (0,6)
Viudo	1 (0,3)
Nivel educativo (N = 334)	
Educación primaria incompleta	23 (6,9)
Educación primaria completa	47 (14,1)
Educación secundaria	202 (60,5)
Educación superior	62 (18,6)
Fuente de ingresos (N = 229)	
Sin ingresos	53 (23,1)
Asalariado	105 (45,9)
Jubilado o pensionista	2 (0,9)
Autónomo	47 (20,5)
Otros	22 (9,6)
**Perfil obstétrico**	
Primer embarazo (N = 334)	115 (34,4)
Embarazo planificado (N = 333)	123 (36,9)
Embarazo deseado (N = 227)	169 (74,4)
Embarazo de alto riesgo (N = 334)	262 (78,4)
Aborto espontáneo/muerte fetal/nacimiento de feto muerto previo (N = 334)	84 (25,1)
Antecedentes de violencia sexual y/o física (N = 334)	35 (10,5)
Tratamiento psiquiátrico (N = 229)	51 (22,3)
Experiencia de parto anterior (N = 209)	
Extremadamente positiva	27 (12,9)
Muy positiva	28 (13,4)
Positiva	70 (33,5)
Normal	42 (20,1)
Negativa	23 (11,0)
Muy negativa	8 (3,8)
Extremadamente negativa	11 (5,3)

N: frecuencia absoluta; %: frecuencia relativa en porcentaje.

La puntuación media del W-DEQ(A) fue de 59,65 (DE ± 23,41), con puntuaciones que oscilaron entre 5 y 136. La Tabla 2 presenta la estratificación de los niveles de miedo, indicando que se observó tocofobia (W-DEQ(A) ≥ 100) en el 4,8 % de las participantes.

**Tabla 2:** Frecuencia de las puntuaciones de miedo al parto entre las mujeres embarazadas de la Maternidad del Complejo Hospitalario de la Universidade Federal do Paraná – Curitiba, Brasil, entre 2021 y 2023.

Variable	N (%)
Puntuación de miedo (N = 334)	
Baja	52 (15,6)
Moderada	150 (44,9)
Alta	88 (26,3)
Muy alta	44 (13,2)
Puntuación de miedo (N = 334)	
< 100	318 (95,2)
≥ 100	16 (4,8)

N: frecuencia absoluta; %: frecuencia relativa en porcentaje.

La comparación entre los distintos niveles de miedo al parto y las motivaciones específicas relacionadas con ese miedo reveló que los niveles más altos de miedo al parto se asociaban con una mayor proporción de ciertas motivaciones relacionadas con el miedo. En concreto, los temores relacionados con el pánico, la pérdida de control, el dolor intenso del parto, el parto prolongado y la incertidumbre sobre la duración del parto fueron más frecuentes entre las mujeres con niveles altos y muy altos de miedo, lo que explica la significación estadística general observada (p < 0,001) (Tabla 3).

**Tabla 3:** Comparación entre las puntuaciones de miedo al parto y las motivaciones relacionadas con el miedo entre las mujeres embarazadas de la Unidad de Maternidad del Complejo Hospitalario de la Universidade Federal do Paraná – Curitiba, Brasil, entre 2021 y 2023.

Variable		N (%)	Bajo N (%)	Moderado N (%)	Alto N (%)	Muy altoN (%)	Valor de p*
¿Miedo a entrar en pánico?	Si	144 (43,1)	8 (15,4)	46 (30,7)	57 (64,8)	33 (75,0)	<0,001
¿Miedo a perder el control de la situación?	Si	185 (55,4)	13 (25,0)	81 (54,0)	57 (64,8)	34 (77,3)	<0,001
¿Miedo a que la vida sexual se vea afectada negativamente después del parto?	Si	49 (14,7)	1 (1,9)	20 (13,3)	19 (21,6)	9 (20,4)	0,009
¿Miedo al dolor del parto?	Si	241 (72,6)	25 (49,0)	106 (70,7)	72 (82,8)	38 (86,4)	<0,001
¿Miedo a las intervenciones médicas (como la episiotomía o la anestesia)?	Si	170 (51,0)	19 (36,5)	70 (47,0)	56 (63,6)	25 (56,8)	0,009
¿Miedo a sufrir una laceración perineal?	Si	195 (58,7)	20 (38,5)	90 (60,0)	53 (61,6)	32 (72,7)	0,005
¿Miedo a una hemorragia?	Si	257 (77,2)	36 (69,2)	114 (76,5)	72 (81,8)	35 (79,5)	0,374
¿Miedo a que me tengan que hacer una cesárea de urgencia?	Si	144 (43,5)	17 (33,3)	61 (40,7)	45 (52,3)	21 (47,7)	0,128
¿Miedo a que el equipo obstétrico (médicos y enfermeras) me trate mal?	Si	269 (80,8)	35 (67,3)	120 (80,5)	77 (87,5)	37 (84,1)	0,030
¿Miedo al parto prematuro?	Si	214 (64,1)	28 (53,8)	92 (61,3)	64 (72,7)	30 (68,2)	0,110
¿Miedo a tener un parto muy largo?	Si	261 (78,4)	29 (55,8)	113 (75,3)	79 (90,8)	40 (90,9)	<0,001
¿Miedo a que mi hijo sufra de alguna manera?	Si	304 (91,3)	42 (80,8)	137 (91,3)	84 (96,5)	41 (93,2)	0,015
¿Miedo a morir durante el parto?	Si	223 (67,2)	25 (50,0)	100 (66,7)	67 (76,2)	31 (70,4)	0,018
¿Miedo a que mi esposo/pareja me vea en estado de pánico?	Si	31 (13,5)	3 (7,7)	8 (8,2)	13 (21,0)	7 (23,3)	0,030
¿Miedo a perder la compostura (“perder el control”)?	Si	50 (22,0)	3 (7,7)	16 (16,5)	23 (37,7)	8 (26,7)	0,001
¿Miedo a no tener la garantía de que podré tener un parto vaginal?	Si	79 (34,8)	6 (15,4)	31 (32,0)	31 (50,0)	11 (37,9)	0,004
¿Miedo a no darme cuenta de una situación anormal?	Si	150 (66,1)	18 (47,4)	59 (60,8)	50 (80,6)	23 (76,7)	0,002
¿Miedo a no saber cuánto durará el trabajo de parto?	Si	129 (57,3)	12 (30,8)	53 (54,1)	38 (65,5)	26 (86,7)	<0,001
¿Miedo a que mi esposo/acompañante no pueda acompañarme durante el parto?	Si	127 (55,5)	17 (43,6)	57 (58,2)	38 (61,3)	15 (50,0)	0,292
¿Miedo a que mi esposo/acompañante se comporte mal?	Si	28 (12,2)	2 (5,1)	8 (8,2)	14 (22,6)	4 (13,3)	0,023
¿Miedo a que se rompa la bolsa amniótica antes de la hospitalización?	Si	78 (34,1)	10 (25,6)	30 (30,6)	23 (37,1)	15 (50,0)	0,145
¿Miedo a que el parto ocurra antes de llegar al hospital?	Si	143 (62,4)	17 (43,4)	65 (66,3)	39 (62,9)	22 (73,3)	0,045
¿Miedo a ser hospitalizada antes del inicio del parto?	Si	65 (28,4)	9 (23,1)	27 (27,5)	18 (29,0)	11 (36,7)	0,660

N: frecuencia absoluta; %: frecuencia relativa en porcentaje;

*Test Chi cuadrado de Pearson o Test exacto de Fisher.

## DISCUSIÓN

Este estudio reveló que el miedo al parto era frecuente entre las participantes. Aunque una proporción considerable de mujeres presentaba un miedo alto o muy alto, predominaban los niveles moderados de miedo. Las principales preocupaciones de las participantes estaban relacionadas con el dolor, la pérdida de control y la incertidumbre respecto a la duración y la evolución del parto. Estos hallazgos refuerzan la idea de que el miedo al parto es un fenómeno multifactorial influenciado tanto por factores psicológicos como obstétricos, y resaltan la importancia de abordar las necesidades emocionales e informativas durante la atención prenatal.

En el presente estudio, el miedo al dolor del parto fue uno de los temores relacionados con el parto más frecuentes y significativos, lo que sugiere que la percepción y la anticipación del dolor desempeñan un papel central en la experiencia del miedo al parto^(21)^. Estos resultados concuerdan con estudios previos, que indican que la catastrofización del dolor es relevante para comprender el miedo al parto. El miedo al dolor y a tener una baja tolerancia al dolor se han descrito como causas comunes del miedo al parto, y sirven como predictores de la intensidad del dolor percibida durante el parto^(22,23)^.

En este contexto, el dolor influye en el comportamiento de las mujeres embarazadas a través del miedo, convirtiéndose en la fuente de otras emociones aversivas y preocupaciones relacionadas con el parto. A diferencia del embarazo, que permite una adaptación gradual a los cambios durante un largo período, el parto es un evento que induce cambios abruptos e intensos, simbolizados por la intensidad del dolor y la imprevisibilidad, lo que conduce a angustia, ansiedad e inseguridad^(24)^. El miedo al dolor del parto está fuertemente asociado con el miedo al dolor en general, independientemente de la paridad, y es una de las razones más comunes para solicitar una cesárea^(8)^. Además, el miedo puede afectar el funcionamiento cognitivo, aumentar la reactividad psicofisiológica e influir en cómo las mujeres interpretan la información y perciben el dolor del parto y sus consecuencias^(22)^.

En este estudio, las participantes expresaron de manera significativa el miedo a entrar en pánico, a perder la compostura y a perder el control de la situación. Estos miedos pueden reflejar una falta de confianza en el proceso del parto y la incapacidad de regular su propio comportamiento durante el parto^(9,25)^.

El miedo a un parto prolongado y a no saber cuánto durará también fueron hallazgos significativos en el presente estudio. Dichos miedos pueden explicarse por la percepción de imprevisibilidad y falta de control durante el parto, factores que también contribuyen a la preferencia de las mujeres por la cesárea electiva, para evitar el dolor y la pérdida de dignidad^(26)^.

El presente estudio identificó el miedo a que el niño sufra y a que no se garantice un parto vaginal como miedos relevantes relacionados con el parto entre las participantes. Se reportaron hallazgos similares en un estudio con 325 mujeres nulíparas en Arabia Saudita^(27)^, que encontró una asociación entre el miedo al parto y la incapacidad de dar a luz o de dañar o causar sufrimiento al bebé. Además, otro estudio encontró que el miedo de las mujeres a no poder dar a luz también puede estar relacionado con la falta de confianza en el equipo obstétrico, el miedo a su propia competencia y el miedo a perder el control^(9)^.

El miedo a morir durante el parto también fue un hallazgo relevante, en consonancia con estudios previos en los que fue el tipo de miedo más común experimentado durante el embarazo, el parto y el posparto^(9,28,29)^. Dichos miedos pueden reflejar preocupaciones relacionadas con la seguridad materna, la confianza en la atención y la vulnerabilidad percibida durante el parto.

El miedo a ser maltratada por el equipo obstétrico también se identificó como prominente entre las participantes. Este resultado concuerda con un estudio realizado con 100 mujeres suecas con miedo al parto, en el que el 73 % informó que la razón más común para el miedo era la falta de confianza en el equipo obstétrico^(13)^. De manera similar, un estudio realizado en los Estados Unidos encontró que las quejas a menudo se relacionaban con los proveedores de atención médica, quienes podían agravar los miedos y aumentar la ansiedad durante el proceso del parto^(25)^.

El presente estudio también encontró que el miedo a las intervenciones médicas se asoció significativamente con el miedo al parto. Se reportaron preocupaciones similares en un estudio brasileño reciente con 25 mujeres embarazadas de alto riesgo, en el que la gran mayoría informó de miedo asociado con la atención obstétrica y las intervenciones médicas durante el trabajo de parto y el parto^(30)^. Estos hallazgos resaltan la importancia de la confianza, la comunicación y la atención respetuosa para mitigar los miedos relacionados con el parto.

Las participantes también informaron de manera significativa sobre el miedo a la laceración perineal y a que su vida sexual se viera afectada negativamente después del parto. Estos hallazgos concuerdan con estudios previos, en los que las mujeres embarazadas informaron de miedo a las lesiones del tracto genital^(13,29)^, así como con investigaciones realizadas en Turquía, donde las mujeres temían que las lesiones perineales pudieran afectar su vida sexual en el futuro^(31)^.

En un estudio japonés con 11 primigrávidas, que exploró las dimensiones cualitativas del miedo al parto, dos participantes manifestaron temor a que algo anormal pudiera suceder durante el trabajo de parto, y una participante expresó temor a que su acompañante se comportara de manera inapropiada^(32)^. Estos hallazgos concuerdan con los del presente estudio, en el que el miedo a no darse cuenta de una situación anormal durante el trabajo de parto y a que el acompañante se comportara mal fue significativo.

El miedo a que el esposo o acompañante la viera en estado de pánico y a que el parto ocurriera antes de llegar al hospital fueron hallazgos significativos entre las participantes del presente estudio. No se identificó literatura relevante que abordara estos miedos específicos, lo que sugiere que estos pueden representar aspectos novedosos del miedo al parto.

Los ítems “miedo a la hemorragia”, “miedo a necesitar una cesárea de emergencia”, “miedo al parto prematuro”, “miedo a que el esposo/acompañante no pueda acompañarla durante el parto”, “miedo a que se rompa la bolsa amniótica antes de la hospitalización” y “miedo a ser hospitalizada antes del inicio del parto” no fueron significativos en relación con el miedo muy alto al parto. Esto sugiere que las pacientes tenían menos miedo a situaciones que pudieran ser manejadas por el equipo médico.

El presente estudio destaca la prevalencia, la intensidad y las características de los miedos relacionados con el parto entre las mujeres embarazadas atendidas en un hospital materno público terciario del sur de Brasil. Las preocupaciones predominantes se referían al dolor, la pérdida de control y la incertidumbre, lo que pone de relieve la necesidad de protocolos de detección sistemática e intervenciones individualizadas para apoyar la salud mental y el bienestar materno. La identificación temprana de estos miedos puede mejorar el bienestar psicológico materno, mejorar la preparación para el parto y promover experiencias positivas de parto.

Desde una perspectiva profesional y de salud pública, la integración de estrategias de reducción del miedo en la atención prenatal de rutina podría mejorar la satisfacción materna y fortalecer los resultados generales maternos y neonatales. Dichas estrategias incluyen educación individualizada, información clara y accesible, apoyo emocional y el fomento de la participación activa en la planificación del parto. Además, fomentar la familiaridad con el entorno de la maternidad y generar confianza en el equipo obstétrico puede ayudar a reducir las intervenciones innecesarias.

No obstante, deben reconocerse algunas limitaciones. El presente estudio se llevó a cabo en un centro regional de referencia para embarazos de alto riesgo, lo que limitó el reclutamiento de mujeres con perfiles de bajo riesgo durante la recolección de datos. Como resultado, la muestra está compuesta predominantemente por embarazos de alto riesgo dentro del sistema de salud pública, lo que puede restringir la generalización de los hallazgos a las mujeres que reciben atención privada o aquellas con embarazos de bajo riesgo. Además, dada la considerable diversidad sociocultural que existe en Brasil, los datos recolectados en un solo centro de salud pueden no ser representativos de la población en general. Por otra parte, la administración del cuestionario durante el tercer trimestre —un período comúnmente asociado con síntomas de ansiedad intensificados— junto con la recolección de datos durante la aplicación de la legislación estatal que permite cesáreas electivas sin indicación médica, puede haber influido en las respuestas de las participantes, lo que podría haber amplificado o mitigado los niveles de miedo reportados.

Cabe destacar que la extensión y la complejidad del cuestionario también pueden haber contribuido a reducir el tamaño de la muestra, lo que plantea dudas sobre su viabilidad en entornos no de investigación. Además, dado que los datos se recolectaron mediante cuestionarios autoadministrados, es posible que se haya producido una falta de respuesta a ciertos ítems, lo que ha dado lugar a una pérdida sustancial de datos para algunas variables y podría haber limitado una evaluación más exhaustiva de los factores asociados al miedo al parto. Los estudios futuros deberían considerar estrategias para mejorar la exhaustividad de los datos en los instrumentos autoadministrados y garantizar una evaluación más sólida de las variables.

Además, este estudio no incluyó un enfoque cualitativo para explorar las dimensiones subjetivas de los miedos de las mujeres, lo que podría haber proporcionado una comprensión más profunda de sus experiencias emocionales. Las investigaciones futuras deberían tener como objetivo identificar preguntas clave que permitan una evaluación rápida del miedo al parto durante las consultas prenatales de rutina, y desarrollar herramientas que traduzcan los hallazgos en indicadores prácticos capaces de orientar las intervenciones clínicas de una manera más accesible y aplicable.

El uso del cuestionario W-DEQ(A), desarrollado originalmente en 1998, en el contexto obstétrico actual y sin una validación formal para la población embarazada brasileña, también representa una limitación de este estudio. Aunque se llevó a cabo un riguroso proceso de traducción y re-traducción y el instrumento demostró una consistencia interna aceptable en la presente muestra (alfa de Cronbach = 0,71), este procedimiento no sustituye a un proceso completo de adaptación cultural y validación. Por lo tanto, los resultados deben interpretarse con cautela. Se justifican estudios futuros para validar el W-DEQ(A) en el contexto brasileño y evaluar su desempeño psicométrico en entornos obstétricos contemporáneos.

A pesar de estas limitaciones, el estudio ofrece contribuciones significativas al campo al reforzar la importancia de reconocer el miedo al parto como una preocupación clínica significativa. Destaca la necesidad de que los profesionales de la salud incorporen la atención de salud mental junto con enfoques multidisciplinarios dentro de los servicios perinatales, con el objetivo de mejorar los resultados tanto para las madres como para sus recién nacidos.

## CONCLUSIÓN

El estudio identificó que las mujeres con un miedo al parto muy elevado experimentaban un mayor temor a entrar en pánico, a no saber cuánto durará el parto, a tener un parto muy largo, a perder el control de la situación y al dolor del parto. Estos hallazgos demuestran que la imprevisibilidad del proceso del parto y la percepción de falta de control sobre la situación son factores clave para intensificar el miedo al parto. Los hallazgos resaltan la importancia de las estrategias de apoyo psicológico e informativo para las mujeres embarazadas, con el objetivo de reducir la ansiedad y fortalecer su confianza en el proceso del parto.

## Data Availability

Todo el conjunto de datos que apoya los resultados de este estudio fue publicado en el propio artículo.
